# Growth Disorders Among 6-Year-Old Iranian Children

**DOI:** 10.5812/ircmj.6761

**Published:** 2014-06-05

**Authors:** Roya Kelishadi, Masoud Amiri, Mohammad Esmaeil Motlagh, Mahnaz Taslimi, Gelayol Ardalan, Reza Rouzbahani, Parinaz Poursafa

**Affiliations:** 1 Child Growth and Development Research Center, Department of Pediatrics, School of Medicine, Isfahan University of Medical Sciences, Isfahan, IR Iran; 2 Social Health Determinants Research Center, Department of Epidemiology and Biostatistics, School of Health, Shahrekord University of Medical Sciences, Shahrekord, IR Iran; 3 Bureau of Family Health, Ministry of Health and Medical Education, Tehran, IR Iran; 4 Department of Pediatrics, Ahvaz Jundishapur University of Medical Sciences, Ahvaz, IR Iran; 5 Bureau of Health and Fitness, Ministry of Education and Training, Tehran, IR Iran

**Keywords:** Body Mass Index, Child, Socioeconomic Factors

## Abstract

**Background:**

Sociodemographic factors are important determinants of weight disorders. National representative studies provide a view on this health problem at national and regional levels.

**Objectives:**

This study aimed to assess the distribution of growth disorders in terms of body mass index (BMI) and height in 6-year-old Iranian children using geographical information system (GIS).

**Materials and Methods:**

In this cross-sectional nationwide survey, all Iranian children entering public and private elementary schools were examined in a mandatory national screening program in 2009. Descriptive analysis was used to calculate the prevalence of underweight, overweight, obesity, and short stature. Then, ArcGIS software was used to draw the figures.

**Results:**

The study population consisted of 955388 children (48.5% girls and 76.5% urban). Overall, 20% of children were underweight, and 14.3% had high BMI, consisted of 10.9% overweight and 3.4% obese. The corresponding figure for short stature was 6.6%; however, these growth disorders were not equally distributed across various provinces.

**Conclusions:**

Our results confirmed unequal distribution of BMI and height of 6-year-old children in Iran generally and in most of its provinces particularly. The differences among provinces cannot be fully explained by the socioeconomic pattern. These findings necessitate a comprehensive national policy with provincial evidence-based programs.

## 1. Background

Body mass index (BMI) and its interpretations about underweight or overweight are major public health concerns worldwide (1-10). Both underweight and overweight (11, 12) are important issues for health policy makers because of their simultaneous presence in the childhood, especially in developing countries. Furthermore, developing countries, like Iran are facing a transition in epidemiological diseases, and nutritional patterns (13). Iran is a vast country, with great socioeconomic and demographic diversity in its provinces; therefore, it is expected to observe a substantial inequality in BMI and height for age index distribution across Iranian provinces.

National representative studies may help us have a view on these health concerns at national and regional levels. Most of the previous studies in Iran have been limited to one city, and thus their results could not generalize to the whole country (14-16); however, there are some national studies, which have considered all provinces of Iran (6-8). Based on the results of these studies, the prevalence of underweight and overweight in different age groups were similar, with more overweight seen in elementary-school students compare to high-school students (7, 8).

## 2. Objectives

To our knowledge, there is no national survey on weight and height status of children in Mediterranean East and North Africa (MENA) using geographic information system (GIS). Therefore, the aim of this study was to assess the distribution of growth disorders in terms of BMI and height by age index distribution across Iranian provinces using GIS. 

## 3. Materials and Methods

The data of this cross-sectional study were collected from a nationwide screening program, conducting regularly on all children entering elementary schools by the Ministry of Health and Medical Education and the Ministry of Education and Training. The study was approved by the Research Ethics Committees of both ministries. All children entering elementary school in Iran were enrolled in this study. In Iran, the elementary education is mandatory; therefore, it was expected that the study population comprised all Iranian children entering public and private elementary schools. During summer 2009 (for three months), 955388 Iranian children entering elementary schools were examined by 5582 skilled health care staff in 823 centers and 712 cities and regions. The examination had two levels: introductory (screening) and diagnostic. In the first level, probable diseases and disorders have been screened (at least in 13 different aspects), and potential patients were referred to verify their possible problems in the second level. The screening level consisted of overall health assessment, including physical examination for general appearance, impaired vision, color blindness, impaired hearing, speech problems, skin and hair problems, enlarged thyroid and lymph nodes, height to age ratio and BMI, decayed teeth, abnormalities in the posture, walking, abdomen, chest, genitourinary, and neurologic systems. It was conducted by general practitioners. Then, the diagnosed patients were referred to specialists for further investigation and consult with their parents. The national Data and Safety Monitoring Board closely supervised the quality control, and assurance of each survey. Training sessions were organized at the national level for focal points of different provinces, and in turn for health care providers at provincial and county levels. All instruments were calibrated, and regularly rechecked during the study. Children's weight and height were measured according to standard protocols. BMI was computed as weight in kilogram divided by the square of height in meter. BMI was categorized into four groups: underweight (less than or equal to 5th percentile), normal weight (between 5th and 85th percentiles), overweight (between 85th and 95th percentiles), and obese (equal to or more than 95th percentile). Short stature was considered as height values of less than 3rd percentile, and values between 3rd and 97th percentiles were considered normal. BMI and height levels were categorized according to the growth charts of the Centers for Disease Control and Prevention (17), which are consistent with Iranian charts (7). To validate the quality of data, the checking process was first conducted at the provincial level weekly and then at the national level monthly. Descriptive analysis was used to determine the prevalence of weight disorders and short stature. To draw the figures, ArcGIS software has been used. The differences between various provinces were examined by chi-square test. The data were analyzed using the Statistical Package for Social Sciences (SPSS) version 18.0 (SPSS Inc., Chicago, IL, USA).

## 4. Results

**Figure 1.**
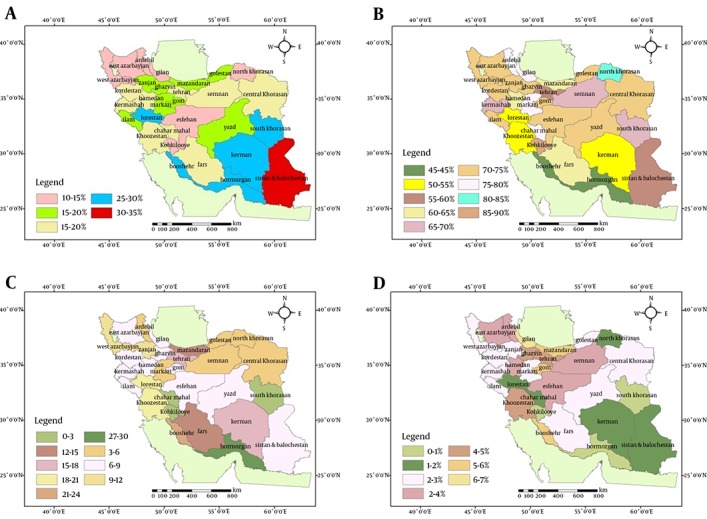
Schematic Distribution of Different Categories of Body Mass Index (BMI) in 6-Year-Old Children Across Iran Provinces A) BMI 5th Percentile, B) BMI 5th-85th Percentile, C) BMI 85th-95th Percentile, D) BMI 95th Percentile. % reflects percentile.

Figure 1 shows the distribution of BMI percentiles across different provinces. Overall, the distribution of underweight and overweight percentages was not similar in all provinces. Sistan and Baluchistan province in the South-East had the highest percentage (30-35 %) of underweight children, followed by Bushehr, Hormozgan, Kerman, South Khorasan, and Lorestan provinces with 25- 30% of underweight children. Isfahan, Kohkilooye, North Khorasan, Ardebil, West Azerbaijan, and East Azerbaijan provinces had at least 10-15% of underweight children. Obesity was documented in 6-7% of children in Gilan, Booshehr and Qom provinces.

 On the other hand, 1-2% of children were obese in Sistan and Baluchistan, Kerman, North Khorasan, Chahrmahal and Bakhtiari and Lorestan provinces; the corresponding figure was 0-1% in Hormozgan, South Khorasan, and Kohkilooye provinces. Table 1 presents the differences among various provinces. Overall, 20% of children were underweight, and 14.3% had high BMI, consisted of 10.9% overweight and 3.4% obese. 

**Figure 2.**
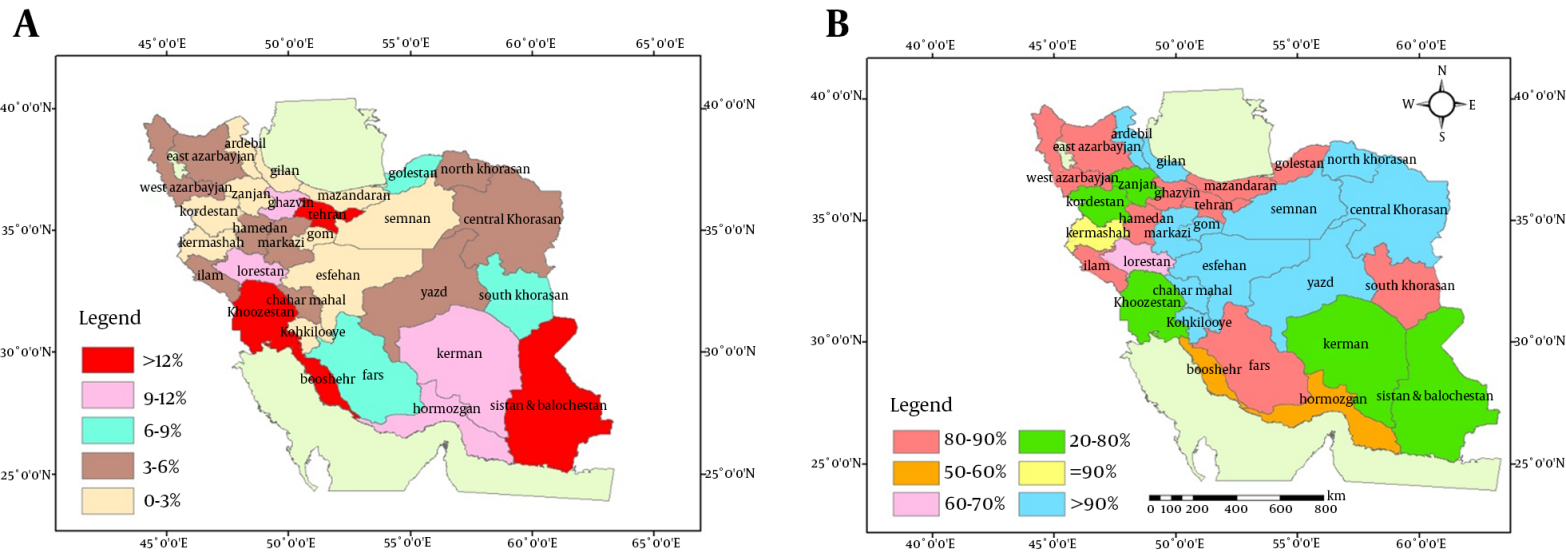
Schematic Distribution of Height for Age Index of 6-Year-Old Children Across Iranian Provinces A) Height for age (3rd percentile), B) Height for age (3rd -97th percentile). % reflects percentile.

Figure 2 demonstrates the distribution of height for age percentiles across different provinces. Likewise, the distribution of height for age percentage was not similar in all provinces. In some provinces such as Tehran, Khuzestan, Sistan and Baluchistan, and Bushehr provinces, the mean height of more than 12% of children was below the 3rd percentile. Overall, 6.6% of children had short stature.

**Table 1.** Distribution of Indices/Percentiles Among Different Provinces ^a^

Characteristics, %	Number of Provinces	P Value ^b^
**BMI ( > 95th percentile)**	-	P > 0.05
0-1	3	
1-2	1	
2-3	10	
3-4	5	
4-5	3	
5-6	7	
6-7	1	
**BMI (95th percentile)**	-	P > 0.05
0-1	3	
1-2	5	
2-3	10	
3-4	5	
4-5	2	
5-6	3	
6-7	1	
**BMI (85-95th percentile)**		P > 0.05
0-3	2	
3-6	6	
6-9	11	
9-12	2	
12-15	2	
15-18	1	
18-21	2	
21-24	1	
27-30	1	
**BMI (5-85th percentile)**	-	P > 0.05
40-45	2	
50-55	3	
55-60	3	
60-65	1	
65-70	3	
70-75	10	
75-80	4	
80-85	1	
85-90	3	
**BMI (5th percentile)**		P > 0.05
10-15	7	
15-20	8	
20-25	9	
25-30	5	
30-35	1	
**BMI (< 5th percentile)**	-	P > 0.05
10-15	7	
15-20	8	
20-25	9	
25-30	5	
30-35	1	
**Height (3rd percentile)**	-	P > 0.05
0-3	10	
3-6	9	
6-9	3	
9-12	4	
> 12	4	
**Height/Age (3rd percentile)**	-	P > 0.05
0-3	9	
3-6	9	
6-9	4	
9-12	4	
> 12	4	
**Height/Age (97th percentile)**	-	P > 0.05
0-3	3	
3-6	12	
6-9	3	
9-12	2	
12-15	2	
15-18	1	
18-21	1	
> 21	1	
= 21	1	
28	1	
31	1	

^a^ Abbreviation: BMI, body mass index.

^b^ Chi-square test.

## 5. Discussion

To the best of our knowledge, the present study is the first of its kind in MENA, providing information on BMI and height for age index from the entire population of children at school entry using GIS. We confirmed substantial differences in the regional distribution of BMI and height for age index, which is consistent with other nationwide studies (3, 18-21).

In spite of diversity in socioeconomic status of people living in different provinces, the observed differences among various parts of the country cannot be fully explained by the socioeconomic pattern of each province. Therefore, this study could not document the socioeconomic determinants of growth disorders. As an obvious assumption, it seems logical to say that the provinces with the more prevalence of very low or very high BMI/height for age index were economically deprived; however, this prevalence was low in some provinces with a similar socioeconomic situation.

The irregular distribution of BMI and height for age index across Iranian provinces does not follow the socioeconomic distribution. Both more- and less-developed provinces were in the 3rd percentile of height for age index and 5th percentile of BMI. These findings suggest a considerable inequality in the distribution pattern of BMI and height for age index in Iranian provinces. The first explanation for this inequality would be the different nutritional patterns among Iranian provinces. However, in recent decades, Iran has experienced a big improvement in maternal and child nutritional status (22), therefore, the role of other factors such as the different patterns of micronutrient distribution, might be more important.

Another explanation for the observed inequality might be related to the ethnic differences. The various Iranian provinces have their own ethnic diversity. However, these differences are more socioeconomic-related than ethnic-related, because even in the provinces with a mixture of ethnic groups, the distribution of BMI and height for age index was similar to the pattern of provinces with one ethnic group.

The most important strength of our study is its nationwide coverage of the whole population of near one million Iranian children, and using GIS to assess the geographical distribution. The other strength is the comparison of BMI and height for age index pattern of children across Iranian provinces. However, the study had its own limitations too. The main limitation of this study is its cross-sectional nature; moreover, we used the study report to present the GIS figures, because we did not have access to the raw data of the survey. In addition, because of this large study population, documenting details of socioeconomic and potential lifestyle determinants of growth disorders was not possible. Also, we could not assess other anthropometric measures such as waist circumference and subcutaneous fat due to inability of collecting these data in such a huge population. Finally, our suggestion on ethnic differences is just based on living area, which does not exactly reflect details of ethnicity-related variances, as many ethnicities in Iran have merged into other ethnicities. This study confirmed unequal distribution of BMI and height for age index across Iranian provinces. These results confirm the necessity of a comprehensive surveillance system and a centralized data registry for Iranian children. Regarding the various growth disorders across different Iranian provinces, future studies could determine the details of sociocultural background and ethnic differences behind that. Furthermore, dietary and physical activity patterns of children (provided by provincial evidence-based programs) are essential for national policy making.
